# Pro-inflammatory S100A8 Protein Exhibits a Detergent-like Effect on Anionic Lipid Bilayers, as Imaged by High-Speed AFM

**DOI:** 10.1021/acsami.4c18749

**Published:** 2024-12-26

**Authors:** Rimgailė Tamulytė, Ieva Baronaitė, Darius Šulskis, Vytautas Smirnovas, Marija Jankunec

**Affiliations:** † Institute of Biochemistry, Life Sciences Center, Vilnius University, Saulėtekio av. 7, Vilnius, LT-10257, Lithuania; ‡ Institute of Biotechnology, Life Sciences Center, Vilnius University, Saulėtekio av. 7, Vilnius, LT-10257, Lithuania

**Keywords:** S100A8, neurodegeneration, membrane solubilization, atomic force microscopy, electrochemical impedance spectroscopy

## Abstract

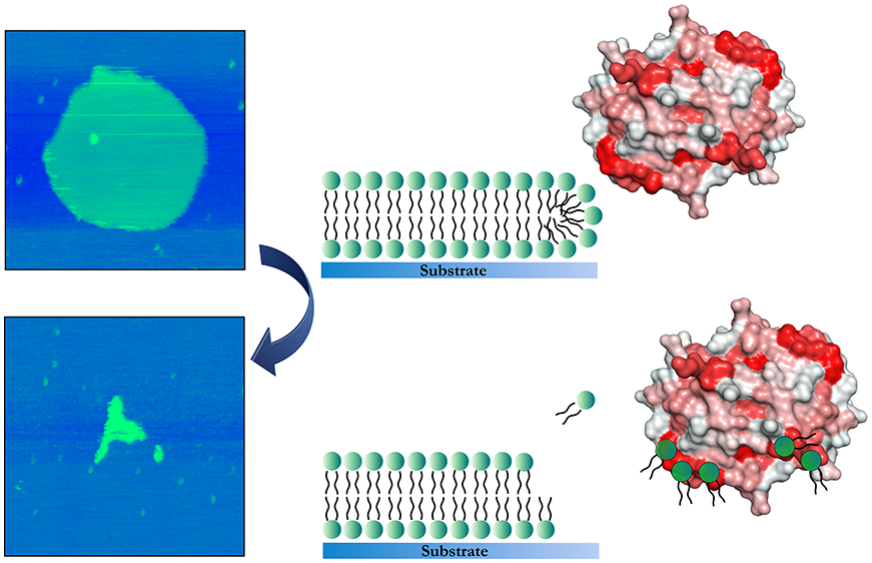

Neuronal cell death induced by cell membrane damage is one of the major hallmarks of neurodegenerative diseases. Neuroinflammation precedes the loss of neurons; however, whether and how inflammation-related proteins contribute to the loss of membrane integrity remains unknown. We employed a range of biophysical tools, including high-speed atomic force microscopy, fluorescence spectroscopy, and electrochemical impedance spectroscopy, to ascertain whether the pro-inflammatory protein S100A8 induces alterations in biomimetic lipid membranes upon interaction. Our findings underscore the crucial roles played by divalent cations and membrane charge. We found that apo-S100A8 selectively interacts with anionic lipid membranes composed of phosphatidylserine (PS), causing membrane disruption through a detergent-like mechanism, primarily affecting regions where phospholipids are less tightly packed. Interestingly, the introduction of Ca^2+^ ions inhibited S100A8-induced membrane disruption, suggesting that the disruptive effects of S100A8 are most pronounced under conditions mimicking intracellular compartments, where calcium levels are low, and PS concentrations in the inner leaflet of the membrane are high. Overall, our results present a mechanistic basis for understanding the molecular interactions between S100A8 and the plasma membrane, emphasizing S100A8 as a potential contributor to the onset of neurodegenerative diseases.

## 1 Introduction

Inflammation arises from both innate and acquired immune responses and is partially mediated by S100 proteins, which are primarily derived from myeloid cells.^1^ Under physiological conditions, these small (10–14 kDa) calcium-binding proteins play roles in a range of intracellular and extracellular functions, including the regulation of protein phosphorylation, cell proliferation, neurite extension, cytoskeletal dynamics, cell migration, and intracellular calcium homeostasis.^2^ However, during chronic inflammation, levels of several S100 proteins are massively elevated, suggesting that these proteins could serve as potential diagnostic markers for various inflammatory diseases.^3^ To date, 25 members of this protein family have been identified in humans,^4^ primarily existing as homo- or heterodimers.^5^

S100 proteins are characterized by two calcium-binding EF-hand motifs: a classical C-terminal EF-hand with a high-affinity Ca^2+^ binding loop, and a S100-specific N-terminal ‘pseudo EF-hand’ exhibiting a lower calcium-binding affinity.^4^ Structural comparisons of the crystal structures of apo (calcium-free) and holo (calcium-bound) S100 proteins demonstrate that, with the exception of S100A10, Ca^2+^ binding typically causes helix rearrangements, exposing a hydrophobic surface that is crucial for target protein recognition.^6^ Besides Ca^2+^, several S100 proteins can bind Mg^2+^, Zn^2+^, or Cu^2+^, usually leading to subtle conformational changes.^7,8^ These conformational changes modulate their functional properties, affecting their affinity for interaction partners and promoting oligomerization.^9^ Although S100 proteins are found both intracellularly and extracellularly, they lack the transmembrane domains or signal peptides required for the classical Golgi-mediated secretion pathway. As a result, alternative, yet not fully understood mechanisms mediate their release.^10^ Given the crucial role of Ca^2+^ in numerous signaling pathways, it is plausible that calcium-dependent binding to the cell membrane could be an initial step toward membrane translocation.

In our study, we explored the S100A8 protein, which has the ability to form homodimers or heterodimers with its partner S100A9, the latter known as calprotectin.^11^ Recent research highlights the complexity and key roles of the S100A8 protein in various processes related to the progression of neurodegenerative diseases. Notably, S100A8 has been identified as a key pathogenic gene, playing a crucial role in the transition from mild cognitive impairment to Alzheimer’s disease.^12^ Extracellularly, S100A8 accumulates and forms nonfibrillar aggregates in the hippocampi of Alzheimer’s disease mouse models that overproduce amyloid-beta peptide (Aβ), such as Tg2576 and TgAPParctic mice.^13^ Additionally, S100A8 interacts with the receptor for advanced glycation end products (RAGE),^14^ expressed in microglia, neurons, and astrocytes.^15^ RAGE facilitates the transport of Aβ across the blood–brain barrier, contributing to its accumulation in the brain.^16^ Intracellularly, S100A8 activates MAP kinase and NF-κB signaling pathways,^17^ implicated in mediating the toxic and pro-inflammatory effects of Aβ both *in vitro* and *in vivo*.^18,19^

Considering that the integrity of plasma membranes is crucial for maintaining biological processes and regulating intracellular calcium levels, and that any disruption can trigger apoptosis in neuronal cells,^20^ the interaction of S100 proteins with the lipid bilayer has garnered significant research attention. Despite a high degree of structural similarity,^21^ S100 proteins exhibit diverse interactions with membranes, primarily through their calcium-dependent binding to specific lipid components, such as arachidonic acid,^22^ or membrane-associated proteins.^23^ Studies on model membranes have demonstrated that apo-S100A8/A9 interacts with zwitterionic bilayers and partially inserts into the outer leaflet.^24^ Additionally, S100G associates with zwitterionic detergent dodecyl phosphocholine (DPC) micelles in the absence of calcium, but specific interactions with individual DPC molecules occur only upon Ca^2+^ binding.^25^ S100A12 interacts with both zwitterionic and negatively charged membranes in its apo- and holo-forms,^9^ whereas S100A10 has a greater affinity for negatively charged polar head groups than zwitterionic ones.^26^ Furthermore, S100A9 was found to accumulate on the anionic phospholipid bilayers. In addition to electrostatic effects, phase-sensitive interactions between S100A9 and lipids were observed, with prior research indicating that apo-S100A9 disrupts raft-like and gel-like domains.^27^ However, despite their potential significance in neurodegeneration studies, the mechanisms underlying the S100A8-lipid interactions remain unknown.

In this study, we aimed to investigate the unresolved question of how S100A8 might affect the lipid bilayer in relation to cell membrane damage and neuronal loss. We used tryptophan fluorescence and circular dichroism to examine how the binding of divalent cations alters the secondary structure of S100A8. We then assessed the effect of S100A8 on the lipid bilayer integrity using electrochemical impedance spectroscopy (EIS) and a calcein leakage assay. Lastly, we employed high-speed atomic force microscopy (HS-AFM) to investigate the impact of S100A8 on the membrane morphology with high spatiotemporal resolution. Our combined approach revealed that apo-S100A8 specifically targets negatively charged lipid bilayers, resulting in membrane disruption through a detergent-like mechanism. Importantly, our methodology allowed for the detection of protein–lipid interactions at anticipated pathological concentrations, thereby enhancing our understanding of S100A8’s involvement in the progression of neurodegenerative diseases.

## 2 Results and Discussion

### 2.1 Determination of Lipid Binding Potential

**Figure 1:**
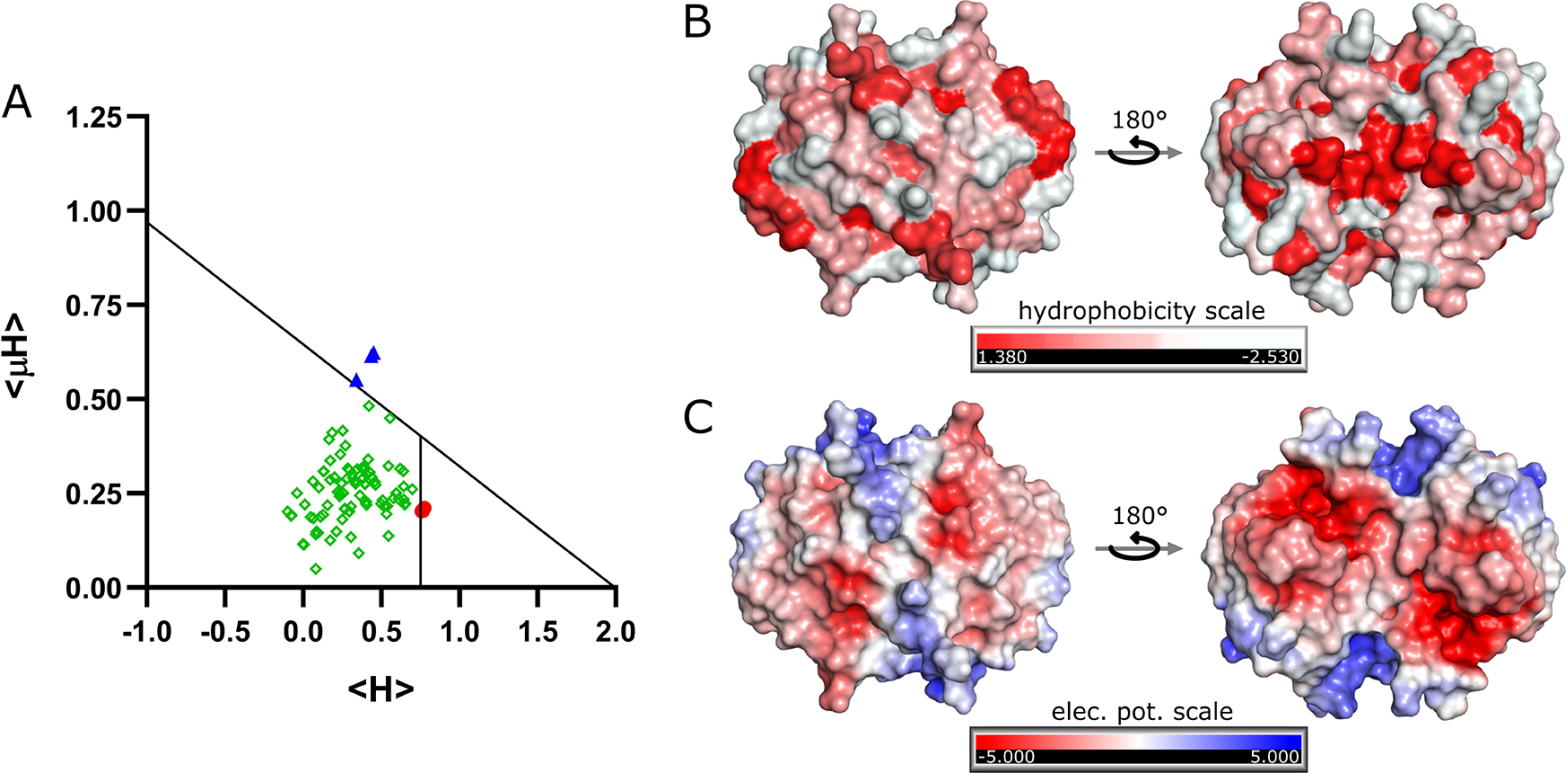
Lipid binding potential of the S100A8 protein. Panel (A) displays the Eisenberg plot generated using data from the Heliquest server. The *x*-axis represents the mean hydrophobicity (⟨H⟩) values, and the *y*-axis shows the hydrophobic moment (⟨μH⟩). Segments identified as globular (highlighted in green squares), surface-seeking (highlighted in blue triangles), and transmembrane (highlighted in red circles) are shown. Panel (B) shows a surface representation of the predicted apo-S100A8 homodimer structure, with hydrophobicity indicated by color. Amino acids are color-coded according to the Eisenberg hydrophobicity scale:^31^ white represents the most hydrophilic regions, while red highlights the most hydrophobic areas. Panel (C) depicts the surface electrostatic potential, with positive charge shown in blue and negative charge in red. The 3D structure of the apo-S100A8 homodimer was predicted using the AlphaFold 3 server.^30^

The S100A8 protein, with a theoretical isoelectric point (pI) of 6.6, has the potential to bind lipid membranes at a physiological pH through electrostatic interactions. To identify potential structure-determined interactions with lipid barriers, the complete sequence of S100A8 was initially submitted to the Web server Heliquest.^28^ To classify whether a region of a protein sequence corresponds to a globular, surface-seeking, or transmembrane segment, we utilized the Eisenberg plot methodology as previously described.^29^ As demonstrated in the Eisenberg plot (Figure 1A), the majority of 18-residue windows correspond to globular protein structures; however, the region AA64–83, with mean hydrophobicity (⟨H⟩) values above 0.75, might indicate a potential transmembrane domain. The AA1–20 region, with significantly high hydrophobicity moment (⟨μH⟩) and ⟨H⟩ values, presumably will act as a surface-seeking protein segment. Following an additional discrimination factor (D)-based analysis, which incorporates both ⟨μH⟩ and net charge (z), we examined four α-helical regions of S100A8 (Table1). However, only Helix IV (AA68–86) had a D value greater than 0.68, suggesting that this α-helix has the potential to bind membranes and membrane-mimicking surfaces. Figure 1B depicts the apo-S100A8 homodimer structure as predicted by the AlphaFold 3 server,^30^ with surface-exposed hydrophobic sites indicated.

**Table 1:**
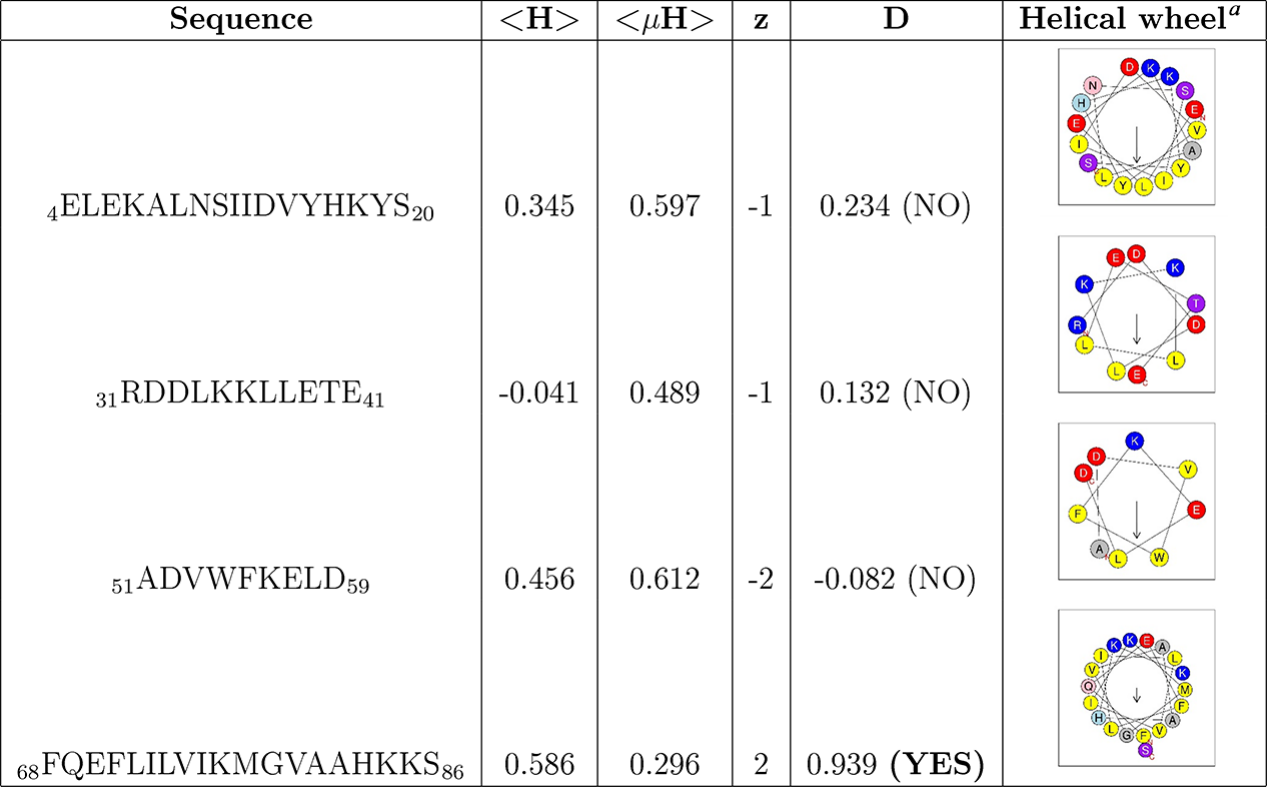
α-Helical Regions of the S100A8 Protein with Calculated Discrimination Factor (D), Mean Hydrophobicity (⟨H⟩), Hydrophobic Moment (⟨μH⟩), and Net Charge (z)

a Helical wheel diagrams were generated using the Heliquest server. In these diagrams, hydrophobic residues are shown in yellow, serine and threonine in purple, basic residues in dark blue, acidic residues in red, asparagine and glutamine in pink, alanine and glycine in gray, histidine in light blue, and proline in green circles. Arrows indicate the direction of the hydrophobic moment. The residue labeled N denotes the N-terminal end of the putative amphipathic helix, and the residue labeled C denotes the C-terminal end.

Notably, while S100A8 carries a slight negative charge (−1.7) at pH 7.4, Helix IV, identified as a potential transmembrane region, exhibits a minor positive net charge (+0.35) under the same conditions (Figure 1C). This positive charge may help mitigate repulsive interactions with negatively charged bilayers, thereby facilitating membrane binding. Additionally, a membrane-seeking segment in the N-terminal half of the S100A8 protein, specifically within EF-hand I, is abundant in basic amino acids. This basic region is conserved across S100 family members,^32^ emphasizing its role in regulating the functional activity of these proteins.

### 2.2 The Effect of apo-S100A8 on Membrane Barrier Properties

**Figure 2:**
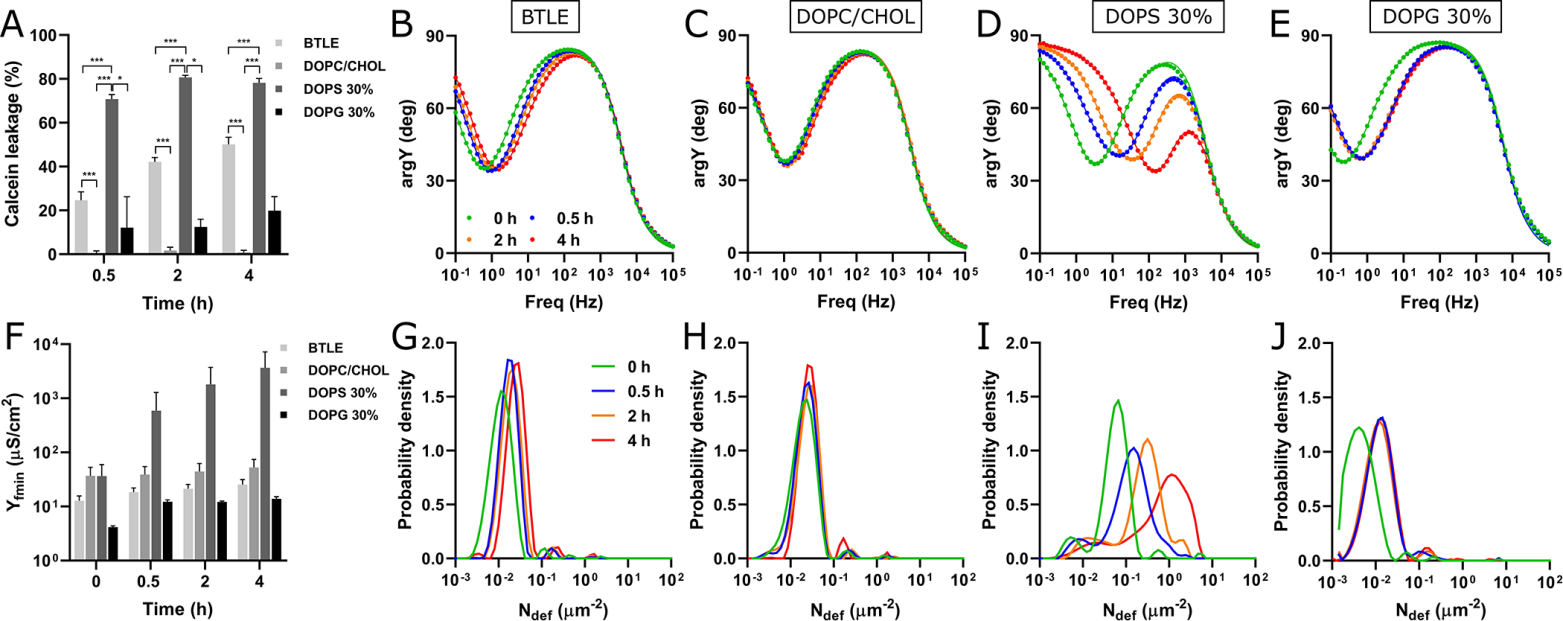
Impact of apo-S100A8 on membrane barrier properties. Panel (A) presents a bar plot illustrating calcein leakage, while panels (B–E) display admittance phase (argY) versus frequency (Freq) (Bode) plots. Circles represent experimental data, and solid lines indicate fitted EIS spectra at different time points: 0 h (green), 0.5 h (blue), 2 h (orange), and 4 h (red). Panel (F) depicts the S100A8-induced alterations in membrane admittance values at the frequency minimum (Y_fmin_). Panels (G–J) show the defect density distribution functions of the lipid membranes with the compositions indicated above. Calcein leakage was measured in a 10 mM HEPES/NaOH buffer at pH 7.4 and 37 °C, while EIS experiments were conducted at 22 °C. The protein concentration was maintained at 10 μM. The lipid composition labeled as DOPS 30% corresponds to DOPC/DOPE/DOPS/CHOL (2/3/3/2), while DOPG 30% denotes DOPC/DOPE/DOPG/CHOL (2/3/3/2). Statistical significance is indicated by * (*p* ≤ 0.05) and *** (*p* ≤ 0.001).

The standard protocol for determining a protein’s ability to disrupt lipid bilayers involves measuring the release of fluorescent dye from liposomes.^33^ We investigated the interaction of S100A8 with liposomes of varying biophysical properties: those composed of natural brain total lipid extract (BTLE), zwitterionic liposomes containing dioleoylphosphatidylcholine (DOPC) and cholesterol (CHOL), and anionic liposomes made from DOPC, CHOL, dioleoylphosphatidylethanolamine (DOPE), and either dioleoylphosphatidylglycerol (DOPG) or dioleoylphosphatidylserine (DOPS). The 4 h incubation period was selected for evaluation as it effectively captures the progressive effects of S100A8 on membrane integrity while preserving sufficient membrane structure for subsequent computational data analysis. Following a 4-h incubation with S100A8 protein, the greatest liposome leakage (77 ± 2%) was observed in negatively charged membranes composed of DOPC/DOPE/DOPS/CHOL (2/3/3/2) (Figure 2A). In the case of BTLE, the permeabilization process was slightly slower, resulting in a smaller extent of dye leakage (54 ± 3%) after 4 h. In contrast, S100A8 did not interact with zwitterionic DOPC/CHOL (6/4) liposomes to cause subsequent membrane permeabilization. Representative curves illustrating the kinetics of S100A8-dependent calcein leakage, including data for extended incubation times (17 h), are presented in Figure S1.

To determine whether S100A8 specifically interacts with DOPS or merely needs an anionic headgroup to permeabilize membranes, we also tested lipid vesicles incorporating DOPG. The release of the fluorescent dye calcein was generally lower in DOPG-containing liposomes compared to that in those containing DOPS. Although both DOPS and DOPG have a net charge of −1 at physiological pH, their charge distributions differ. PS contains a negatively charged phosphate group interacting with serine residue which has both positive (NH^3+^) and negative (COO^–^) charges. In contrast, the headgroup of phosphatidylglycerol (PG) remains uncharged, with its negative charge originating from the phosphate group binding to glycerol within the polar headgroup. Consequently, PS-containing vesicles are more anionic than PG-containing ones at physiological pH.^34^ Additionally, PS occupies a larger area (0.97 nm^2^ per lipid) compared to PG (0.8 nm^2^),^26,35^ which may enhance its interaction with S100A8.

To overcome the limitation of the calcein leakage assay in detecting defects smaller than 1.3 nm,^36^ we employed electrochemical impedance spectroscopy to monitor changes in the dielectric properties of bilayers on a solid support. In the absence of S100A8, Bode plots of phase angle versus frequency exhibited an inflection point centered between 0.2 and 3.2 Hz, depending on the lipid composition of the membrane (Figure 2B-E). We demonstrate that the pristine membranes remained stable throughout the 4 h experimental period (Figure S2). The electrochemical changes induced by S100A8 were both lipid composition- and time-dependent. Throughout the 4 h experimental period, BTLE membranes displayed a continuous, minor shift in the minimum point of the negative impedance phase (*f*_min_), indicating impaired insulation properties of the lipid bilayer and alterations in ion movement across it. In contrast, the immediate effects of S100A8 on DOPC/DOPE/DOPG/CHOL membranes were evident during the initial 0.5 h, after which the *f*_min_ value stabilized and remained constant. However, alterations in the dielectric properties of DOPC/DOPE/DOPG/CHOL bilayers may also result from intrinsic rearrangements within the membrane structure, as comparable changes in *f*_min_ position were observed in the control membranes during the corresponding 0.5-h period. Remarkably, the most significant effect was observed in the anionic DOPC/DOPE/DOPS/CHOL membrane, where the *f*_min_ shifted nearly 2 orders of magnitude toward higher frequencies over the 4-h incubation period. The extent of membrane damage was further assessed by evaluating the admittance value at the *f*_min_ point (Y_fmin_), which correlates with the density of defects within the membrane.^37^ As illustrated in Figure 2F, the increase in *f*_min_ was accompanied by a corresponding 100-fold increase in Y_fmin_ for the DOPC/DOPE/DOPS/CHOL membranes during the same 4-h incubation period. In contrast, no significant changes were observed in the dielectric properties of the DOPC/CHOL membranes upon exposure to S100A8.

To gain a more detailed understanding of the S100A8-induced defect distribution across the membrane surface, we utilized a computational methodology developed by Valincius et al.^38^ Natural defects in the pristine membranes were uniformly distributed across all lipid compositions and were characterized by a single peak in the distribution curves (Figure 2G-J). The most significant increase in the local defect density (N_def_) was observed in negatively charged DOPC/DOPE/DOPS/CHOL membranes upon exposure to S100A8. Additionally, the presence of multiple peaks at different N_def_ values suggests that the protein induces a highly heterogeneous defect distribution. An exponential increase in global defect density was also noted over time, rising from 0.2 μm^–2^ in pristine membrane to 2.4 μm^–2^ after 4 h of exposure to S100A8. In contrast, the increase in global defect densities within other studied membrane compositions over the same 4 h period following S100A8 exposure was relatively minor (Figure S3).

### 2.3 Effects of S100A8 Concentration and DOPS Levels

**Figure 3:**
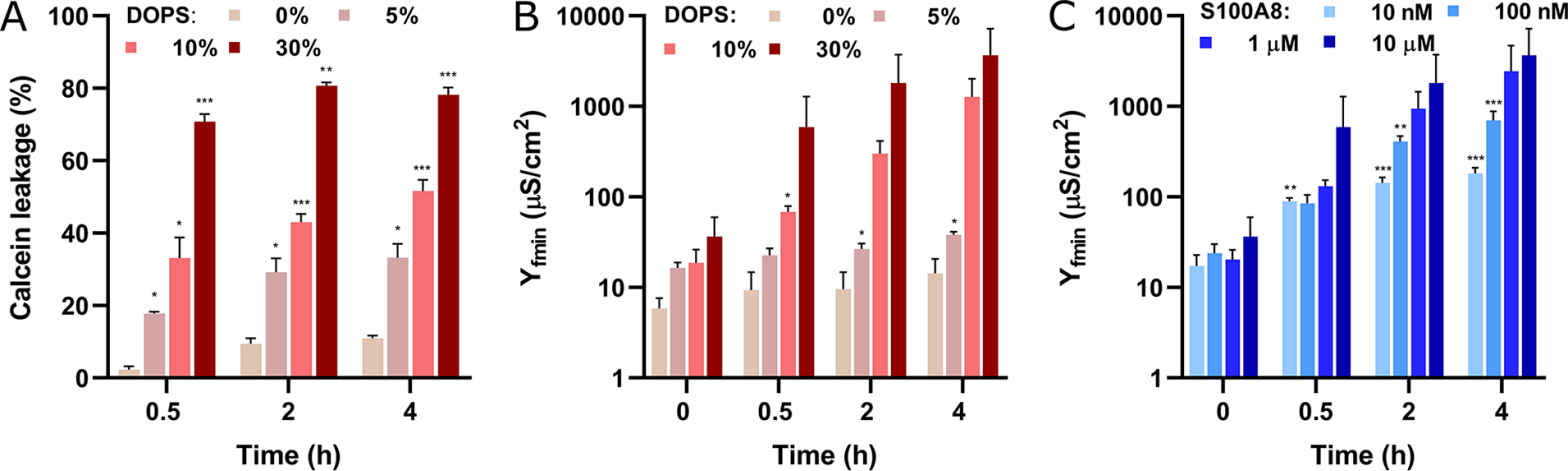
S100A8 disrupts membranes in a DOPS level- and concentration-dependent manner. Bar plots (A) and (B) show calcein leakage and EIS data, respectively, for DOPC/DOPE/DOPS/CHOL membranes treated with 10 μM S100A8. Panel (C) depicts the effect of S100A8 concentrations (10 nM to 10 μM) on the admittance of DOPC/DOPE/DOPS/CHOL (2/3/3/2) membranes. Calcein leakage was measured in 10 mM HEPES/NaOH buffer at pH 7.4 and 37 °C, while EIS experiments were conducted at 22 °C. Statistical significance is indicated by * (*p* ≤ 0.05), ** (*p* ≤ 0.01), and *** (*p* ≤ 0.001). Statistical significance for calcein leakage data was assessed by comparing membranes with different DOPS formulations to those without DOPS at the same time point, while EIS data were compared across different time points under consistent conditions.

Phosphatidylserine is the most abundant anionic phospholipid, accounting for up to 20% of the total lipid content in brain tissue.^39^ To examine its effect on the membrane-disruptive activity of S100A8, we systematically adjusted DOPS concentrations by proportionally altering the levels of DOPC. When the DOPS concentration was reduced from 30% to 20%, membrane permeability after a 4 h incubation remained unchanged (77 ± 2 and 77 ± 4%, respectively) (Figure S4). Liposomes with lower DOPS concentrations (10% or 5%) were significantly less affected by S100A8, resulting in dye releases of 33 ± 5 and 18 ± 1%, respectively, during the initial 0.5 h. An increase in calcein leakage was observed over the following 4 h, reaching 52 ± 3 and 34 ± 4% for liposomes containing 10% and 5% DOPS, respectively. Notably, S100A8 did not cause significant membrane permeabilization in the DOPS-free liposomes (Figure 3A). These results were confirmed by EIS data, which revealed that the increase in admittance magnitude was directly correlated with the concentration of anionic lipids, with the most significant loss of integrity observed in membranes containing 30% DOPS (Figure 3B).

Notably, bilayers containing 10% DOPS (Figure 3B) were more susceptible to S100A8-induced disruption compared with BTLE membranes (Figure 2F), despite their comparable PS content. This observation underscores the importance of factors beyond surface charge, such as lipid packing and membrane phase behavior, in modulating S100A8 activity. BTLE and DOPC/CHOL (6/4) membranes, which are known to adopt a liquid-ordered state,^27^ likely exhibit enhanced mechanical stability, making them more resistant to S100A8-mediated destabilization. In contrast, the more disordered DOPS-enriched bilayers, characterized by reduced cholesterol content, are inherently more susceptible to perturbation by S100A8.

The pronounced affinity of S100A8 for PS suggests that PS may play a pivotal role in mediating the protein’s activity, with potential broader biological implications. In healthy neural cells, PS is predominantly found in the cytoplasmic leaflet of the membrane. However, under conditions such as oxidative stress, PS is translocated to the outer leaflet, signaling cellular apoptosis–a hallmark of Alzheimer’s disease.^40^ Since the secretion pathways of S100A8 remain unclear, we hypothesize that lipid remodeling and the transbilayer movement of PS may facilitate the translocation of S100A8 to the neural cell surface, similar to other PS-binding proteins such as annexin V.^41^ This translocation might occur either through direct binding of S100A8 to PS or through interactions with PS-bound annexin V, which has been shown to associate with S100 family proteins such as S100A9.^42^ Such PS-mediated movement could contribute to the extracellular accumulation and aggregation of S100A8, a phenomenon observed in neurodegenerative contexts.^13^ Additionally, we propose that the increased secretion of S100A8 by immune cells during neuroinflammation^11^ may contribute to enhanced neuronal membrane disruption, particularly when PS is exposed on the cell surface. Moreover, PS is prevalent in the outer leaflet of extracellular vesicle (EVs) membranes, which are involved in the intercellular transfer of amyloid-like proteins throughout the brain.^43^ Studies indicate that S100A8 promotes Aβ aggregation within EVs,^44^ with its preferential binding to PS on the EVs surface likely enhancing this effect.

Examining the correlation between S100A8 concentration and its disruptive potential on membranes containing 30% DOPS, we tested protein concentrations ranging from 10 nM to 10 μM. We observed a continuous increase in membrane admittance with rising S100A8 concentrations (Figure 3C). Notably, even at the lowest concentration of 10 nM, S100A8 induced a statistically significant 11-fold increase in membrane admittance during the 4 h incubation period. Although the exact concentration of S100A8 is not yet fully established, typical levels in the bloodstream range from 47 pM^45^ to 1.9 nM^46^ under physiological conditions. Notably, patients with Alzheimer’s disease exhibit an almost 6-fold increase in S100A8 expression.^47^ Therefore, our methodology demonstrates exceptional sensitivity, enabling the detection of S100A8 activity at concentrations close to the pathological levels. Recognizing the potential of tethered bilayer lipid membranes as sensing elements in impedimetric biosensors^48^ and acknowledging S100A8 as a key marker in various inflammation-related pathologies,^3^ our methodology holds promise for advancing future biosensing applications.

### 2.4 Visualization of the Dynamics of Membrane Disruption

**Figure 4:**
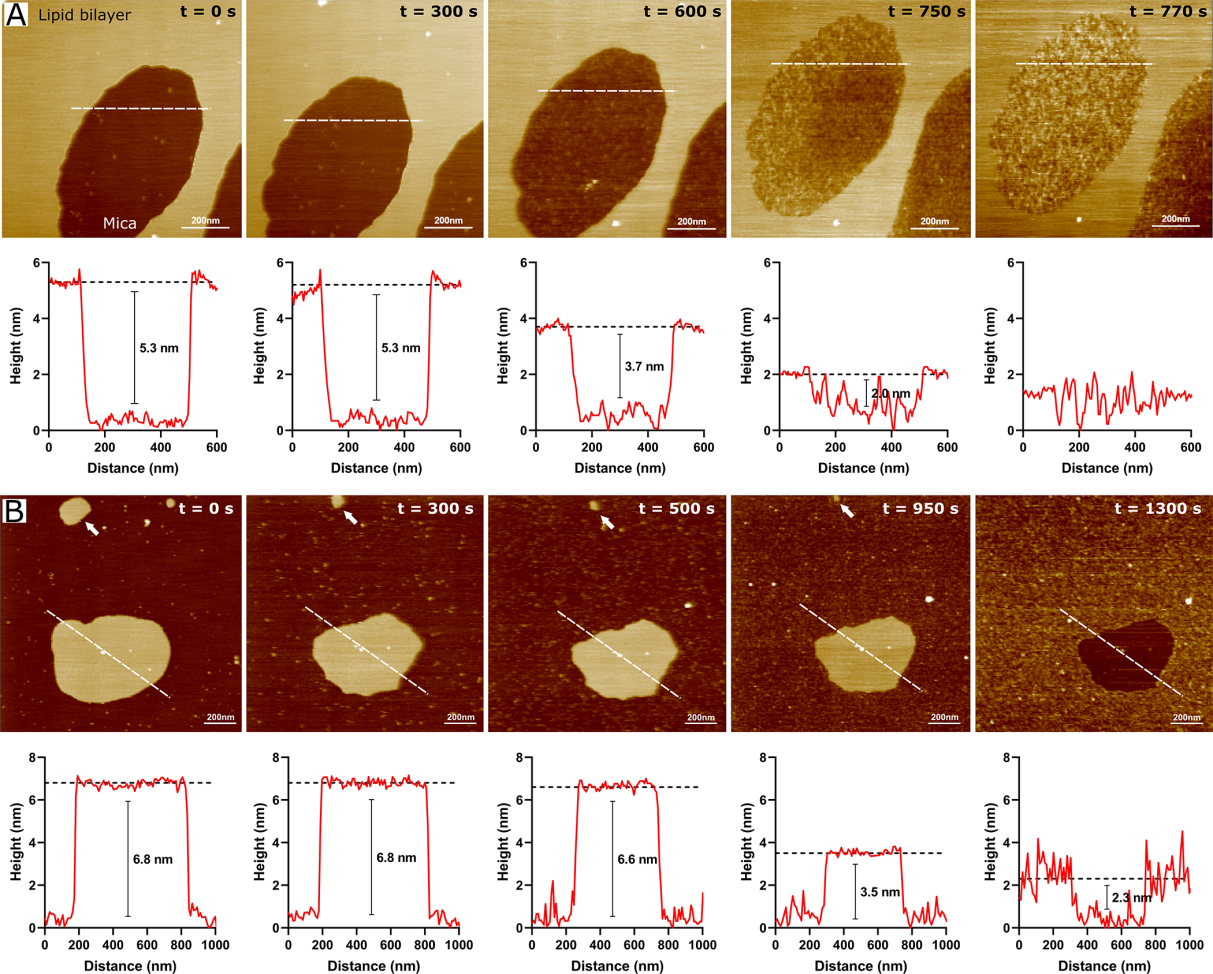
HS-AFM observations of S100A8 protein interactions with BTLE (A) and DOPC/CHOL (6/4) (B) solid-supported lipid bilayers. Successive HS-AFM images, extracted from Videos S1 and S2, show the states before (*t* = 0 s) and after protein injection at the indicated time points. Scale bar: 200 nm. The traces below each image display the height profiles measured along the horizontal white lines. The experiments were conducted in HEPES/NaOH buffer (pH 7.4) with a protein concentration of 10 μM.

We further employed HS-AFM to examine the morphological changes in the membrane induced by S100A8. Unlike conventional atomic force microscopy, HS-AFM enables the assessment of dynamics within biologically relevant time frames, facilitating a real-time analysis of S100A8 activity. The interactions between S100A8 and a model membrane initially composed of BTLE were investigated. HS-AFM images obtained from the recorded video (see Video S1) are presented in Figure 4A. Following vesicle fusion onto the substrate, a region displaying both membrane and mica surfaces was selected. The pristine membrane (bright area at *t* = 0 s) had a flat and smooth surface, with a bilayer thickness of 5.3 nm, consistent with prior reports on the thickness of BTLE membrane.^49^ Following the injection of S100A8 at the initial time point (*t* = 0 s), the protein began to accumulate around the membrane on the negatively charged^50^ mica surface. After 10 min of incubation, S100A8 was present on the substrate, achieving full coverage of the mica within 12.8 min of recording. Notably, no interactions between the protein and the membrane were observed throughout the measurement period. In this instance, the affinity between S100A8 and the planar BTLE bilayer was insufficient to induce membrane disruption.

A similar outcome was observed with the zwitterionic DOPC/CHOL membrane, where S100A8 spread across the mica substrate (Figure 4B). In this instance, the mica surface was fully covered within 15.8 min, and after 21.6 min of incubation, protein accumulation on the substrate led to the appearance of protrusions around the membrane. A representative image of S100A8 deposited on the mica substrate, obtained from Video S2, reveals that the majority of the adsorbed protein particles are spherical, with heights varying between 1.1 and 6.5 nm (Figure S5). To further investigate the structural properties of S100A8, measurements were conducted in air by depositing a freshly thawed protein solution onto the mica surface, followed by air-drying prior to imaging. Under these conditions, the average height of S100A8 was significantly reduced to 0.6 ± 0.2 nm (n = 1134) (Figure S6). This reduction can be attributed to the loss of the hydration shell and protein denaturation during the air-drying process. To provide additional context, we utilized the HullRad Web server^51^ to determine the theoretical dimensions of the S100A8 homodimer based on its AlphaFold 3-predicted structure. The analysis determined an anhydrous diameter of 3.7 nm and a hydrodynamic diameter of 4.7 nm, highlighting the substantial influence of environmental conditions on the protein morphological measurements.

Notably, a direct interaction between the protein and the zwitterionic membrane was observed, as indicated by the complete dissolution of a 215 nm-diameter lipid patch (indicated by white arrows in Figure 4B) within 15.8 min. Over the same period, the surface area of the central membrane patch decreased by approximately 35%. Membrane disruption initiated at the edges, suggesting that defects in the bilayer structure may act as initiation points for protein-induced disruption. This phenomenon has been previously interpreted as membrane dissolution via a detergent-like mechanism, supported by comparable effects observed when planar bilayers were exposed to Triton X-100 above its critical micelle concentration.^52^ Similar membrane disruption, resembling detergent activity, has been reported for antimicrobial peptides,^53,54^ amyloid-β,^55^ phospholipases A2 and D,^56,57^ and prion protein.^58^ Although this phenomenon was observed in model membranes, a comparable disruption could also occur in living cells. In biological contexts, interactions between proteins and cell membranes may generate localized forces that modify membrane structure, resembling the edge effects observed in our experiments, potentially leading to membrane damage *in vivo*.

**Figure 5:**
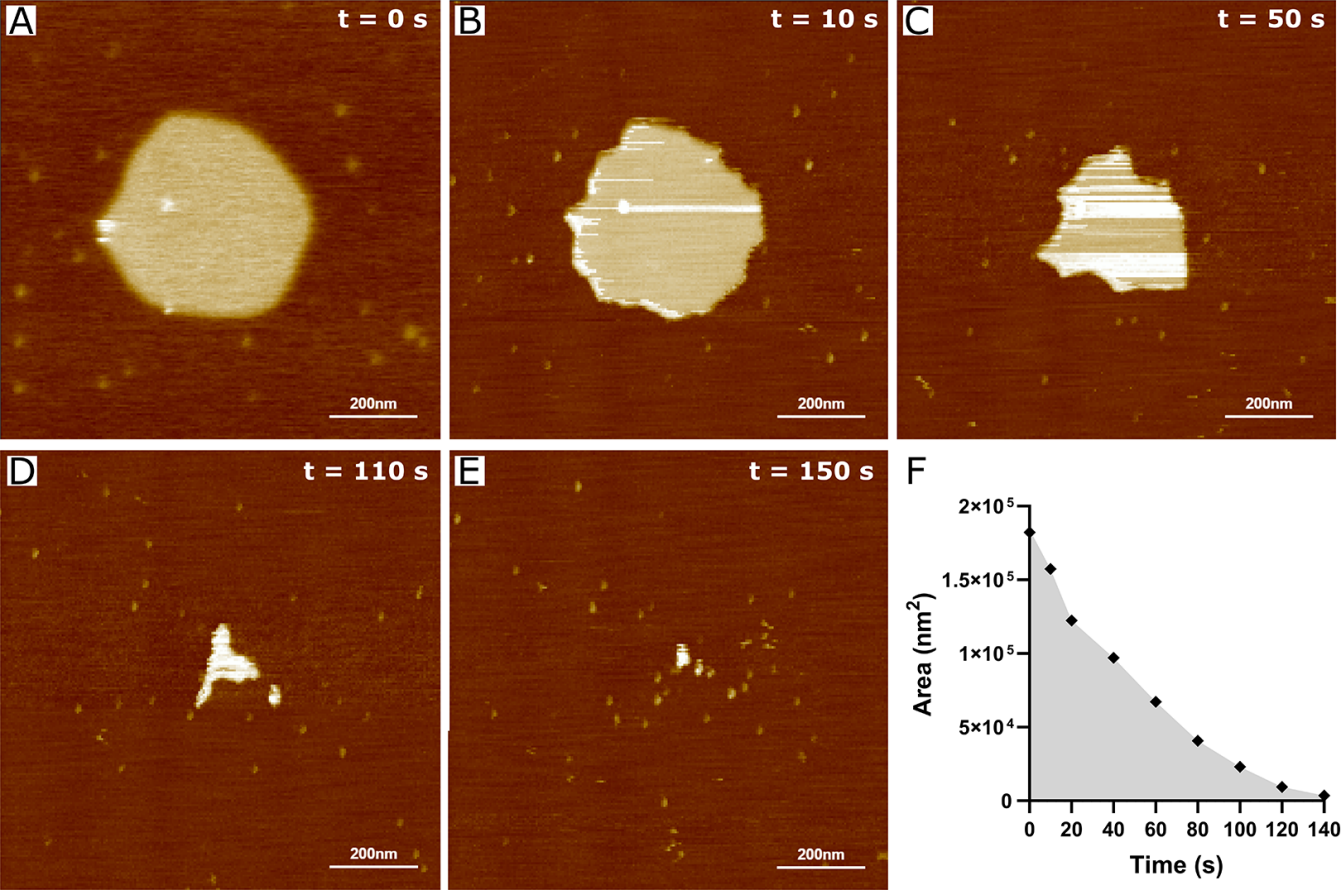
HS-AFM analysis of S100A8 protein interactions with a DOPC/DOPE/DOPS/CHOL (2/3/3/2) solid-supported lipid bilayer. Sequential HS-AFM images, extracted from Video S3, illustrate the state before (A) and after (B–E) protein injection. Scale bar: 200 nm. Panel (F) shows the reduction in membrane surface area over time. The experiment was conducted in HEPES/NaOH buffer (pH 7.4) with a protein concentration of 10 μM.

**Figure 6:**
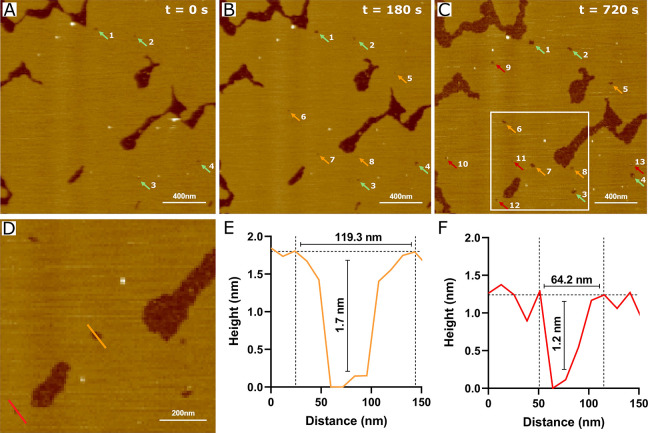
HS-AFM analysis of S100A8 protein interactions with a DOPC/DOPE/DOPS/CHOL (2/3/3/2) solid-supported lipid bilayer. Sequential HS-AFM images, extracted from Video S4, show the state before (A) and after (B, C) S100A8 injection. Panel (D) provides a magnified view of the area indicated in panel (C). Preexisting membrane defects are marked with green arrows, newly formed defects at the 3 min time point are marked with orange arrows, and defects appearing after 12 min are marked with red arrows. Scale bar: 400 nm (200 nm for the magnified view). Panels (E) and (F) display height profiles along the orange and red lines shown in panel (D). The experiment was conducted in HEPES/NaOH buffer (pH 7.4) with a protein concentration of 10 μM.

Distinct protein responses were observed in DOPC/DOPE/DOPS/CHOL membranes, where gradual disruption of the bilayer occurred shortly after the injection of S100A8 (Figure 5A-E, Video S3). Consistent with DOPC/CHOL lipid bilayers, membrane dissolution began at the edges, suggesting a detergent-like effect. However, the membrane disruption occurred in a nonexponential manner (Figure 5F), which contrasts with the EIS data (Figure S3). This difference may be attributed to variations in the membrane structure; the membranes employed in the EIS experiments are highly insulating and exhibit a low defect density. In contrast, the bilayers examined in HS-AFM have more exposed hydrophobic regions, facilitating interactions between S100A8 and the lipids. To compare the data obtained from EIS and HS-AFM experiments, we formed a supported lipid bilayer with extended mica coverage. During a 12 min incubation with the protein, we observed the emergence of new defects and the gradual enlargement of pre-existing ones (Figure 6). Assuming that these defects are circular, the average radius of the newly formed defects was measured to be 25.9 ± 3.9 nm (Table S1). The average depth of these defects was 0.9 ± 0.3 nm, which is smaller than the overall membrane thickness (∼4.4 nm) (Table S2). However, precise depth evaluation is challenging due to tip–sample convolution. Furthermore, consistent with the observations from DOPC/CHOL and BTLE membrane measurements, accumulation on the negatively charged mica substrate was observed following extended incubation periods (12 min and beyond) (Video S4).

A notable aspect of our experiments is the variability in the rate of membrane disruption caused by S100A8. Despite similar protein concentrations in each experiment, a comparison of the results from Figure 5 and Figure 6 reveals that while membrane disruption occurs in both cases, the time required for complete dissolution varies. In the case of the isolated lipid patch, the membrane completely dissolved in under 3 min, whereas with the more continuous bilayer, the membrane was still present after 12 min of measurement. In this context, the crucial factor is not solely the concentration but also the local concentration of the protein near the scanned area. The local concentration cannot be precisely controlled due to various factors, such as the movement of the HS-AFM tip or the distance between the pipet tip and the sample during protein injection. Additionally, the membrane’s morphology may also contribute to the membrane-disruptive potential of the S100A8 protein.

### 2.5 Identification of Divalent Cations-Induced Structural Changes in S100A8

**Figure 7:**
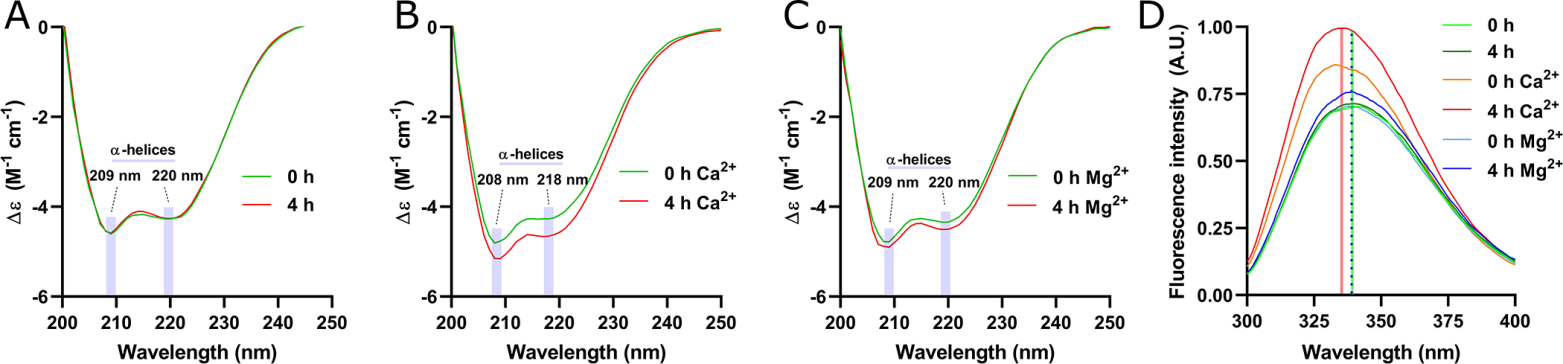
Structural comparison of the S100A8 protein before and after incubation with 2 mM Ca^2+^ or Mg^2+^. Far-UV CD spectra illustrate the structural state of S100A8 following 4 h of incubation without additional cations (A), with Ca^2+^ (B), and with Mg^2+^ (C). Wavelengths corresponding to α-helical signals are highlighted in shaded blue. Intrinsic tryptophan fluorescence emission spectra of S100A8 are depicted in panel (D). The wavelengths of fluorescence intensity maxima after 4 h of incubation without additional cations and with Ca^2+^ are indicated by solid green and red vertical lines, respectively, while the maximum with Mg^2+^ is represented by a blue dotted line. All experiments were conducted in 10 mM HEPES/NaOH buffer, pH 7.4, with a protein concentration of 20 μM

Far-UV circular dichroism (CD) spectroscopy and intrinsic tryptophan (Trp) fluorescence were employed to evaluate the stability of the S100A8 protein and the effects of Ca^2+^ and Mg^2+^ on its secondary structure in solution. The protein predominantly adopts an α-helical conformation, as evidenced by characteristic absorption minima at around 209 and 220 nm in the CD spectrum (Figure 7A). Although our results demonstrate that S100A8 remains stable at room temperature with no significant structural changes over 4 h, its tendency to form spherical aggregates at elevated temperatures and higher protein concentrations has recently been disclosed.^59^ Binding of Ca^2+^ resulted in slight changes in the spectra, with the absorption minima shifting to 208 and 218 nm, respectively (Figure 7B). Furthermore, the molar CD values (Δε) at 208 and 218 nm increased, as illustrated by a change in the Δ_ε218 nm_ value from −4.2 M^–1^ cm^–1^ at 0 h to −4.6 M^–1^ cm^–1^ after 4 h. This increase in CD intensity potentially indicates reorientation of the α-helices,^60^ which may be linked to physiological responses involving the recognition of and interaction with molecular targets, including proteins and other ligands.^61−63^ A similar effect was noted when the protein was incubated with Mg^2+^; over 4 h, the Δ_ε220 nm_ value increased from −4.3 to −4.5 M^–1^ cm^–1^ (Figure 7C).

The intrinsic fluorescence of Trp showed a significant increase of up to 29% in intensity when the protein was incubated with Ca^2+^ (Figure 7D). This increase in fluorescence intensity was accompanied by a blue shift in the emission maximum from 339 to 335 nm, suggesting conformational changes in the protein structure as the Trp_54_ residue becomes more buried within a hydrophobic core. In contrast, the addition of Mg^2+^ ions did not produce a notable shift in the emission maximum wavelength. These findings align with previous reports indicating that Mg^2+^ binding does not significantly alter the conformation of S100 proteins.^8^

### 2.6 The Effect of holo-S100A8 on Membrane Barrier Properties

**Figure 8:**
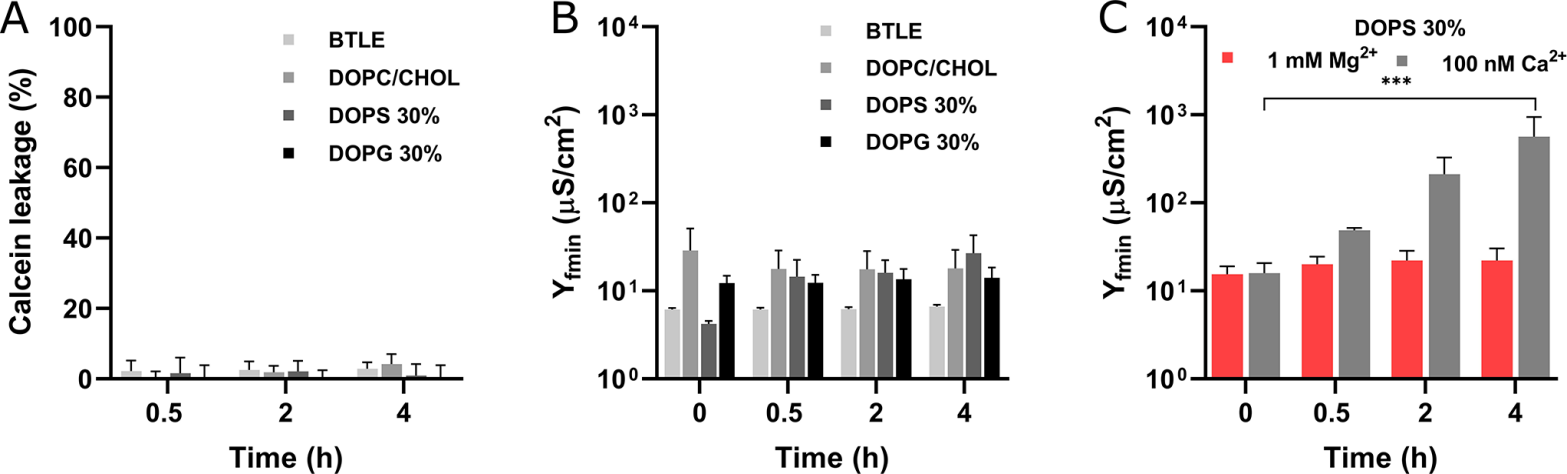
Impact of holo-S100A8 on membrane barrier properties. Bar plots (A) and (B) show calcein leakage and EIS data, respectively, for membranes treated with 10 μM S100A8 in the presence of Ca^2+^. The Ca^2+^ concentration for the EIS experiments was maintained at 1 mM, while for calcein leakage, it was 2 mM. Panel (C) illustrates the changes in membrane admittance induced by S100A8 in the presence of either 100 nM Ca^2+^ or 1 mM Mg^2+^ ions. Calcein leakage measurements were conducted in 10 mM HEPES/NaOH buffer at pH 7.4 and 37 °C, while the EIS experiments were performed at 22 °C. The lipid composition labeled DOPS 30% refers to DOPC/DOPE/DOPS/CHOL (2/3/3/2), whereas DOPG 30% denotes DOPC/DOPE/DOPG/CHOL (2/3/3/2). Statistical significance is indicated by *** (*p* ≤ 0.001).

Previous research has shown that calcium binding alters the conformation of S100 family proteins, exposing hydrophobic groups that promote lipid interactions.^64,65^ To assess the impact of calcium ion binding on the membrane-disruptive activity of S100A8, we tested physiologically relevant calcium concentrations that mimic both extracellular (1–2 mM) and intracellular (100 nM) levels. Our results revealed that higher concentrations of Ca^2+^ (2 mM) significantly inhibited S100A8-induced liposome permeabilization across all lipid compositions studied (Figure 8A). This finding was corroborated by EIS data, which demonstrated that the addition of Ca^2+^ (1 mM) effectively prevented membrane damage, with no statistically significant changes in Y_fmin_ observed over time (Figure 8B).

Divalent cations are recognized for their strong binding affinity to lipid bilayers containing anionic lipids, leading to increased membrane rigidity and organization,^66,67^ changes in lipid headgroup conformation, ordering of lipid tails,^68^ reduced lipid hydration, and neutralization of the membrane’s surface charge.^69^ Therefore, the inhibition of membrane disruption upon calcium binding is likely due to changes in the lipid bilayer rather than alterations in the protein conformation. This is further supported by our observation that Mg^2+^, which does not affect S100A8 conformation (Figure 7D), also inhibited the membrane-disruptive potential of S100A8 (Figure 8C). Notably, the Mg^2+^ concentration used in our experiments (1 mM) aligns with physiological free magnesium levels found in both extra- and intracellular environments.^70^

Importantly, when the Ca^2+^ concentration was reduced to 100 nM, mimicking physiological intracellular levels,^71^ S100A8’s membrane-disrupting activity was only partially diminished. Over a 4-h period, the DOPS-containing bilayer exhibited a 35-fold increase in Y_fmin_ (Figure 8C), which was reduced 3-fold compared to the calcium-free system (Figure 2F). Since S100A8 remains in its apo-form at calcium concentrations as low as 100 nM,^6^ this observation supports the hypothesis that the inhibition of membrane disruption is primarily driven by calcium-induced changes in the lipid bilayer. Nevertheless, the possibility of direct effects of calcium ions on the protein itself cannot be entirely ruled out. While S100A8 exhibits the highest membrane-disruptive activity at low calcium concentrations, which mimic intracellular conditions, we speculate that calcium dysregulation in neurodegenerative diseases may enable S100A8 to exert its membrane-disruptive effects in the extracellular space due to reduced calcium levels.^72^

## 3 Conclusions

Inflammation plays a crucial role in the development of neurodegenerative disorders, with elevated levels of S100 proteins^3^ potentially contributing to plasma membrane damage and subsequent neuronal loss. Recent research has highlighted the role of S100A8 in the progression of neurodegenerative diseases;^12,13^ however, the involvement of the plasma membrane in this process remains unclear. The key finding of this study is the lytic activity of the pro-inflammatory S100A8 protein on artificial lipid bilayers, a phenomenon not previously documented for other members of the S100 protein family. Our results emphasize the role of membrane composition in mediating the toxic effects of S100A8. Specifically, we demonstrate that the extent of membrane disruption correlates directly with anionic DOPS levels and that S100A8-induced disruption follows a detergent-like mechanism, with lipid removal initiating at pre-existing membrane defects. The preferential interaction with PS, the predominant anionic lipid in the inner leaflet of the plasma membrane in neural tissues, underscores the significance of S100A8’s activity on neuronal membranes. Furthermore, our data show that elevated calcium ion concentrations significantly reduce S100A8’s membrane-disruptive activity. Altogether, these findings provide a biophysical rationale for the potential involvement of S100A8 in inflammation-mediated neurodegeneration. However, further research is necessary to fully elucidate the precise mechanisms underlying the interaction between S100A8 and DOPS-containing membranes, including the identification of specific regions within S100A8 that are responsible for these interactions.

## 4 Experimental Procedures

### 4.1 Materials

1,2-Dioleoyl-*sn*-glycero-3-phosphocholine (DOPC), 1,2-dioleoyl-*sn*-glycero-3-phospho-l-serine (sodium salt) (DOPS), 1,2-dioleoyl-*sn*-glycero-3-phosphoethanolamine (DOPE), 1,2-dioleoyl-*sn*-glycero-3-phospho-(1′-rac-glycerol) (sodium salt) (DOPG), cholesterol (CHOL), and brain total lipid extract (BTLE) were purchased from Avanti Polar Lipids (USA). The saline solution contained 0.1 M NaCl and 0.01 M NaH_2_PO_4_ (Roth, Denmark) at pH of 4.5. A 10 mM HEPES buffer (Sigma-Aldrich, Germany) was prepared at pH 7.4. The pH of the buffer was adjusted using a NaOH solution.

The S100A8 expression and purification procedures were carried out as previously described.^59^

### 4.2 Spectroscopy Measurements

All far-UV circular dichroism (CD) spectroscopy measurements were conducted by using a J-815 spectrometer (Jasco, Japan), which was purged with N_2_ and operated at room temperature (22 °C). To investigate the effects of divalent cations on protein conformation, 20 μM solutions of freshly purified S100A8 were prepared in 10 mM HEPES/NaOH buffer (pH 7.4), supplemented with either 2 mM CaCl_2_ or MgCl_2_, or without either cation. Samples were measured immediately after preparation or following a 4 h incubation at room temperature. A quartz cuvette with a path length of 0.1 cm was used for the measurements. CD spectra were recorded from 190 to 260 nm with a 0.5 nm data interval, a 1 nm bandwidth, and a scanning speed of 50 nm/min. Each spectrum represents the average of three scans with the buffer background subtracted. Spectral analysis and visualization were performed using Spectragryph v1.2.16.1 software.^73^

Tryptophan fluorescence spectroscopy experiments were conducted using a CARY Eclipse Fluorescence Spectrophotometer (Varian Inc., USA) with a 0.3 cm path length quartz cuvette at room temperature (22 °C). To minimize interference from the S100A8 tyrosine fluorescence, the excitation wavelength was set to 290 nm. Emission spectra were recorded between 300 and 400 nm with a 5 nm slit width and a dwell time of 0.5 s.

### 4.3 Preparation of Multilamellar and Unilamellar Vesicles

Briefly, the lipids were dissolved in chloroform, and the appropriate volumes of each lipid solution were mixed. The solvent was then evaporated under a stream of N_2_ for 40 min, yielding a thin lipid film. To prepare multilamellar vesicles (MLVs), the dried lipid film was hydrated with 1 mL of saline solution (pH 4.5), achieving a final lipid concentration of 1 mM. The resulting cloudy MLVs suspension was initially ultrasonicated for 1 h. Subsequently, it was extruded 21 times through a mini-extruder (Avanti Polar Lipids Inc., USA) equipped with a 100 nm pore-size polycarbonate membrane, resulting in a clear suspension of large unilamellar vesicles (LUVs). Calcein-loaded LUVs were prepared by rehydrating lipid films in a 10 mM HEPES/NaOH buffer (pH 7.4) containing 60 mM calcein. Free calcein was separated from the calcein-encapsulated LUVs through size-exclusion chromatography by passing the sample through a chromatography column (Lenz Laborglas GmbH & Co. KG, Germany) packed with Sephadex G-50 (Sigma-Aldrich, Germany).

### 4.4 Calcein Leakage Assay

Calcein leakage experiments were conducted using a ClarioStar Plus plate reader (BMG Labtech, Germany) in standard 96-well microplates (Corning Inc., USA). S100A8 protein was introduced into a calcein-loaded LUVs solution, with final concentrations of 100 μM for lipids and 10 μM for the protein, resulting in a protein-to-lipid ratio of 1:10. For experiments with calcium, the Ca^2+^ concentration was maintained at 2 mM. Fluorescence measurements were taken from the bottom of the microplate every 10 min for 17 h, using a 490 nm excitation filter and a 520 nm emission filter, both with an 8 nm band-pass slit. The plate was shaken at 400 rpm between measurements. The calcein leakage assay was performed twice, each time in quadruplicate at 37 °C. At the end of each measurement, maximum leakage was determined by adding Triton-X100 to a final concentration of 1% (v/v). The release of the fluorescent dye was evaluated using the eq 1:
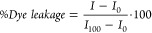
1where *I* and *I*_0_ represent the observed fluorescence intensities of the calcein-loaded LUVs in the presence and absence of the protein, respectively. *I*_100_ corresponds to the 100% leakage value, achieved by treating the calcein-loaded LUVs with 1% Triton X-100. The experimental values were adjusted by subtracting the average control values for all of the lipid compositions.

### 4.5 Preparation of Planar Lipid Bilayers

Gold substrates (with a 10 nm Cr sublayer and a 100 nm Au layer) for electrochemical impedance spectroscopy were prepared on 26 mm × 76 mm glass slides (Paul Marienfeld GmbH & Co. KG, Germany) using a PVD75 magnetron sputtering system (Kurt J. Lesker Co., USA). The freshly coated slides were then immersed in a 0.05 mM solution of tether WC14 (20-tetradecyloxy-3,6,9,12,15,18,22-heptaoxahexatricontane-1-thiol, synthesized in-house) and β-mercaptoethanol (Sigma-Aldrich, USA), mixed in a 3:7 molar ratio in 96.3% ethanol, to form mixed self-assembled monolayers. After incubating for 12 h, the samples were washed with pure ethanol and dried under a nitrogen stream. The tether-functionalized gold films were then placed into an electrochemical setup with 14 independent vials, each with an area of 0.16 cm^2^ and a volume of 280 μL. Tethered bilayer lipid membranes (tBLMs) were formed using the vesicle fusion method.^79^ Briefly, 100 μL of a MLVs solution was added to each vial and incubated for 1 h, followed by washing with 5 mL of MLVs-free 10 mM HEPES/NaOH buffer containing 100 mM NaCl (pH 7.4). The membranes were equilibrated with the HEPES/NaOH buffer for 30 min. Only tBLMs that demonstrated stable dielectric properties were selected for subsequent S100A8 treatment. For experiments involving divalent cations, 1 mM or 100 nM CaCl_2_, or 1 mM MgCl_2_, was added to the vials prior to the protein treatment of the tBLMs.

Solid-supported lipid bilayers (SLBs) for high-speed atomic force microscopy measurements were prepared by the fusion of LUVs onto a mica substrate (grade IV, SPI Supplies, USA). To form the SLBs, a 2 μL droplet of LUVs was placed on freshly cleaved mica, which was glued to a glass rod stage with a diameter of 1.5 mm using nail polish. After incubating in a humid chamber for 10–20 min, the sample was carefully rinsed with liposome-free HEPES/NaOH buffer (pH 7.4).

### 4.6 Electrochemical Impedance Spectroscopy

Electrochemical impedance spectroscopy (EIS) measurements were conducted using the PalmSens4 electrochemical workstation, accompanied by PSTrace 5.9 software (PalmSens BV, Netherlands). The frequency range spanned from 0.1 Hz to 100 kHz, with 10 logarithmically distributed measurement points per decade and a perturbation amplitude of 10 mV. All measurements were performed at room temperature (22 °C) in HEPES/NaOH buffer (10 mM), containing 100 mM NaCl. The measurements were repeated three times for consistency. A Au-coated glass slide served as the working electrode, and a silver wire with a diameter of 1.5 mm, purchased from Sigma-Aldrich (USA), was used as the reference electrode. Prior to use, the silver wire was treated in a 50 mM aqueous solution of iron(III) chloride (Roth, Denmark) for 30 s. The electrode’s validation involved measuring the potential difference in a 3 mM KCl solution (Roth, Denmark) with respect to the confirmed Ag/AgCl reference electrode, also obtained from Sigma-Aldrich (USA). The electrode was considered acceptable for use if the difference fell within the range of 0 ± 20 mV. The admittance of the tBLMs was calculated from the electrochemical impedance spectra using the relation Y_fmin_ = 1/Z_fmin_, where Z_fmin_ represents the impedance modulus measured at *f*_min_.

EIS spectra fitting was performed as described previously.^74,75^ Defect radii (r_0_) and conductance were fixed at 25.9 nm and 50 pS, respectively. Although the exact values for r_0_ might not be precise, they have minimal impact on the overall fit quality and the resulting defect density distribution.^74^ The value for specific resistance of submembrane layer (ρ_sub_) was assumed to be 10^4.5^ Ω, and a thickness (d_sub_) of approximately 1.8 nm, as estimated by neutron reflectometry experiments.^76^ The regularization parameter for all EIS curves was consistently set at −0.9.

### 4.7 High-Speed Atomic Force Microscopy Imaging

All images in this study were captured using a HS-AFM (SS-NEX, RIBM, Japan) operating in amplitude modulation mode, employing ultrashort (8 μm) Si_3_N_4_ rectangular cantilevers (BL-AC10DS-A2, Olympus, Japan) with a radius of 24 nm. These cantilevers had a nominal spring constant of approximately 0.1 N/m and a resonant frequency of around 0.5 MHz in solution. During imaging, the free oscillation amplitude was adjusted to approximately 0.6 nm, with the set-point amplitude set to approximately 80% of the free oscillation amplitude. S100A8 was introduced into the fluid cell filled with 10 mM HEPES/NaOH buffer (pH 7.4) during imaging, reaching a final protein concentration of 10 μM. The resulting changes in the morphology of SLBs were observed at room temperature (22 °C). The scanning speed was set at one frame per 5 s with a resolution of 200 × 200 pixels. Data analysis was performed using NanoLocz 1.20 software.^77^

### 4.8 Bioinformatics Analysis

The Heliquest server^28^ was employed to calculate the mean hydrophobicity (⟨*H*⟩), the hydrophobic moment (⟨*μH*⟩), and the net charge (*z*) for each sequence. The results were plotted on an Eisenberg plot,^31^ where low hydrophobic moment and low mean hydrophobicity values indicate globular protein segments, high ⟨*μH*⟩ and high ⟨*H*⟩ values suggest membrane surface-seeking segments, and low ⟨*μH*⟩ with high ⟨*H*⟩ values indicate segments embedded in membranes. Potential surface-seeking segments are identified as regions on the Eisenberg plot where the hydrophobicity values are above the line defined by ⟨*μH*⟩ = 0.645–0.324⟨*H*⟩. In contrast, potential transmembrane segments are defined as regions with a mean hydrophobicity value exceeding 0.75.

In this analysis, 18-residue windows were used to identify the window with the highest discrimination factor (*D*) for each sequence. The discrimination factor is defined as follows using eq 2:
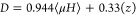
2If *D* exceeds 0.68, the corresponding region can be regarded as a potential lipid-binding helix (see http://heliquest.ipmc.cnrs.fr/ for additional information).

The helical wheel representations were generated using the Heliquest software.^28^ The resulting helical wheel plots were subsequently redrawn and customized. The 3D structure of the apo-S100A8 homodimer was predicted using the AlphaFold 3 server,^30^ based on a protein sequence previously reported in the literature.^78^ The surface representations of the protein were generated using the PyMOL Molecular Graphics System, Version 3.0, Schrödinger, LLC.

### 4.9 Statistical Analysis

Statistical analysis was conducted with GraphPad Prism version 8.4.3 for Windows (GraphPad Software, USA). A significance level (α) of 0.05 was selected for the study. The normal distribution of data was assessed using the Shapiro–Wilk test. For comparing groups with normally distributed data, ANOVA with Bonferroni post hoc test was employed.

## Supporting Information Available

Supporting Information: Data are provided on the stability of the tethered bilayer lipid membranes, time-dependent changes in defect radius and depth induced by the S100A8 protein, along with details on protein size characterization, the kinetics of S100A8-induced calcein leakage, and shifts in global defect densities in lipid bilayers after S100A8 exposure. (PDF)
Video S1: HS-AFM video of the S100A8 interaction with a BTLE lipid bilayer. (MP4)
Video S2: HS-AFM video of the S100A8 interaction with a DOPC/CHOL lipid bilayer. (MP4)
Video S3: HS-AFM video of the S100A8 interaction with a DOPC/DOPE/DOPS/CHOL lipid bilayer (single lipid patch). (MP4)
Video S4: HS-AFM video of the S100A8 interaction with a DOPC/DOPE/DOPS/CHOL lipid bilayer. (MP4)
