# Supplementary material for: Pro-inflammatory S100A8 Protein Exhibits a Detergent-like Effect on Anionic Lipid Bilayers, as Imaged by High-Speed AFM
Source: ACS Appl Mater Interfaces. 2024 Dec 26;17(1):2635–47. doi: 10.1021/acsami.4c18749 (PMC11783366; doi:10.1021/acsami.4c18749)
Supplement: Supplementary file 1 — am4c18749_si_001.pdf [file am4c18749_si_001.pdf]

Supporting Information

Pro-Inflammatory S100A8 Protein Exhibits a Detergent-Like Effect on Anionic Lipid Bilayers, as Imaged by High-Speed AFM

Rimgailė Tamulytė[1,*], Ieva Baronaitė[2], Darius Šulskis[2], Vytautas Smirnovas[2], and Marija Jankunec[1]

[1]Institute of Biochemistry, Life Sciences Center, Vilnius University, Saulėtekio av. 7, Vilnius, LT-10257, Lithuania

[2]Institute of Biotechnology, Life Sciences Center, Vilnius University, Saulėtekio av. 7, Vilnius, LT-10257, Lithuania

*Corresponding Author:

Email: rimgaile.tamulyte@bchi.stud.vu.lt

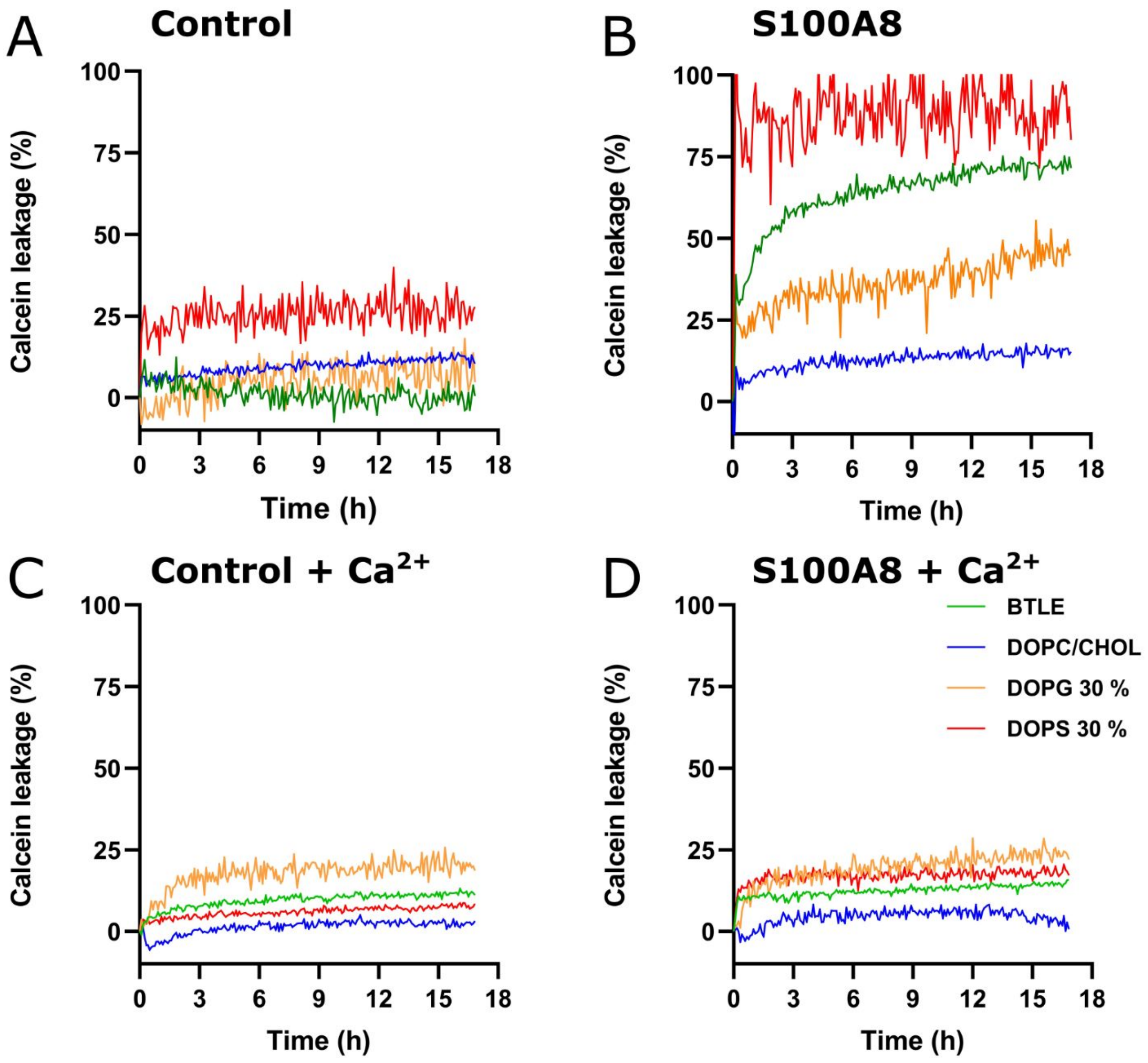

Figure S1: The kinetics of calcein release from liposomes of varying compositions induced by the S100A8 protein. Panels (A) and (C) show spontaneous dye leakage in the absence and presence of Ca^{2+}, respectively. Panels (B) and (D) display S100A8 protein-induced calcein leakage in the absence and presence of Ca^{2+}, respectively. The experiments were conducted in 10 mM HEPES/NaOH buffer at pH 7.4 and 37°C. Protein and total lipid concentrations were maintained at 10 μM and 100 μM, respectively. In experiments with calcium, the Ca^{2+} concentration was standardized to 2 mM. DOPS 30% represents a lipid composition of DOPC/DOPE/DOPS/CHOL (2/3/3/2), while DOPG 30% denotes a composition of DOPC/DOPE/DOPG/CHOL (2/3/3/2).

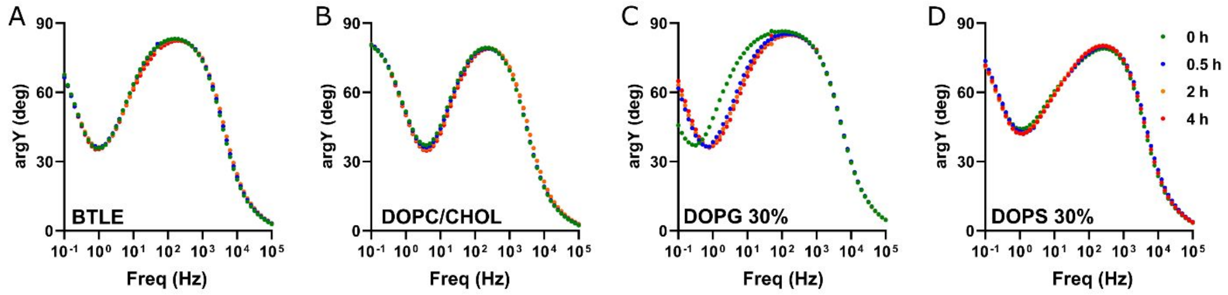

Figure S2: Stability of pristine tethered bilayer lipid membranes (tBLMs) over time. Panels A, B, C, and D display admittance phase (argY) versus frequency (Freq) (Bode) plots for BTLE, DOPC/CHOL, DOPG 30%, and DOPS 30% lipid membranes over a 4-hour incubation period. The lipid composition labeled as DOPS 30% corresponds to DOPC/DOPE/DOPS/CHOL (2/3/3/2), while DOPG 30% refers to DOPC/DOPE/DOPG/CHOL (2/3/3/2).

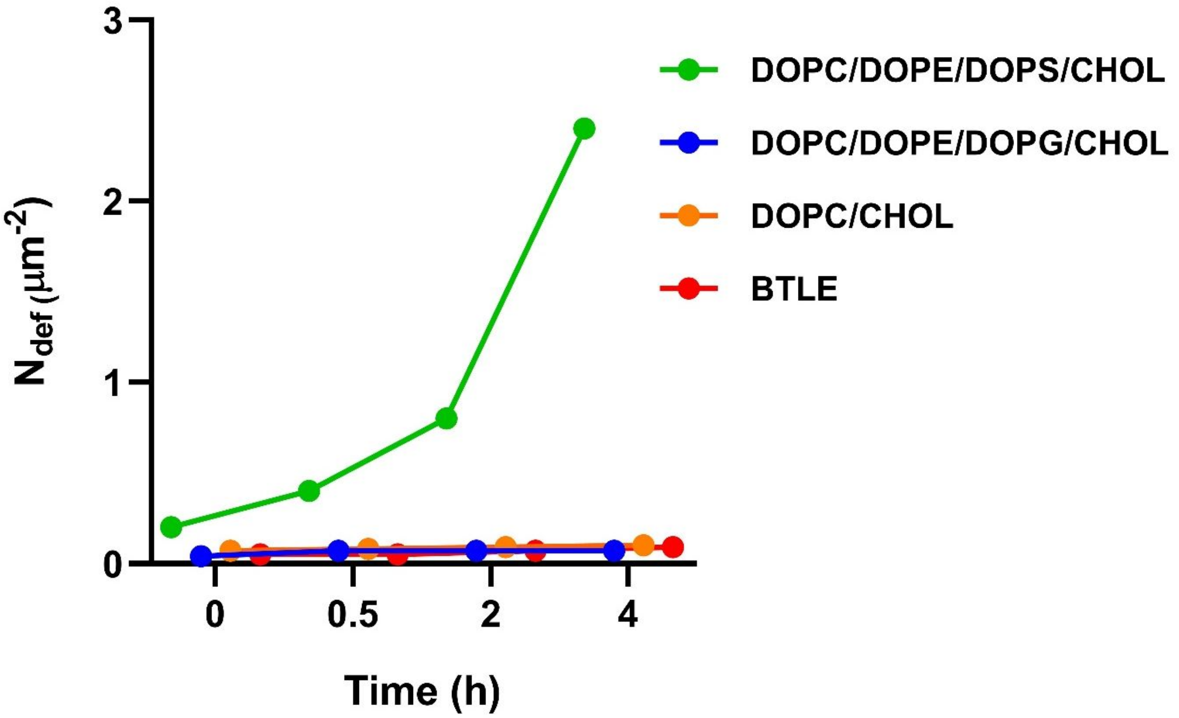

Figure S3: Depiction of global defect density in tethered bilayer lipid membranes (tBLMs) as a function of time. The global defect density was calculated by integrating the area under the defect density distribution curves. The findings show an exponential increase in global defect density in tBLMs composed of DOPC/DOPE/DOPS/CHOL (2/3/3/2) following exposure to 10 µM S100A8. The experiments were performed in 10 mM HEPES/NaOH buffer (pH 7.4) without Ca^{2+}.

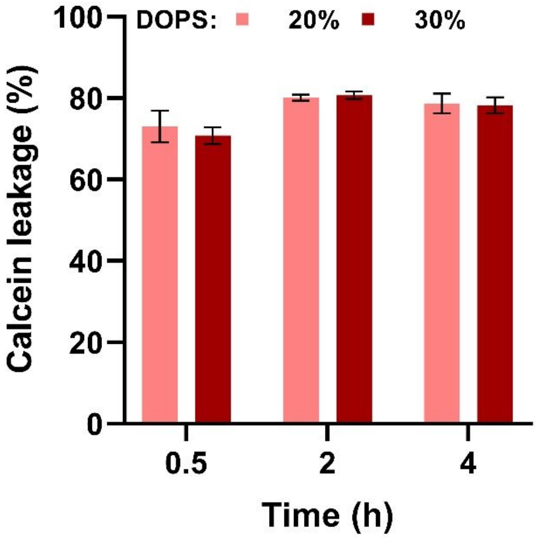

Figure S4: Comparison of membrane disruptive activity of S100A8 in liposomes containing 20 or 30% DOPS. The percentage of calcein leakage was monitored over 4 hours of incubation with the protein. The experiment was conducted in 10 mM HEPES/NaOH buffer at pH 7.4 and 37°C, with protein and total lipid concentrations maintained at 10 µM and 100 µM, respectively. DOPS 30% refers to a lipid composition of DOPC/DOPE/DOPS/CHOL (2/3/3/2), while DOPS 20% denotes a composition of DOPC/DOPE/DOPS/CHOL (3/3/2/2).

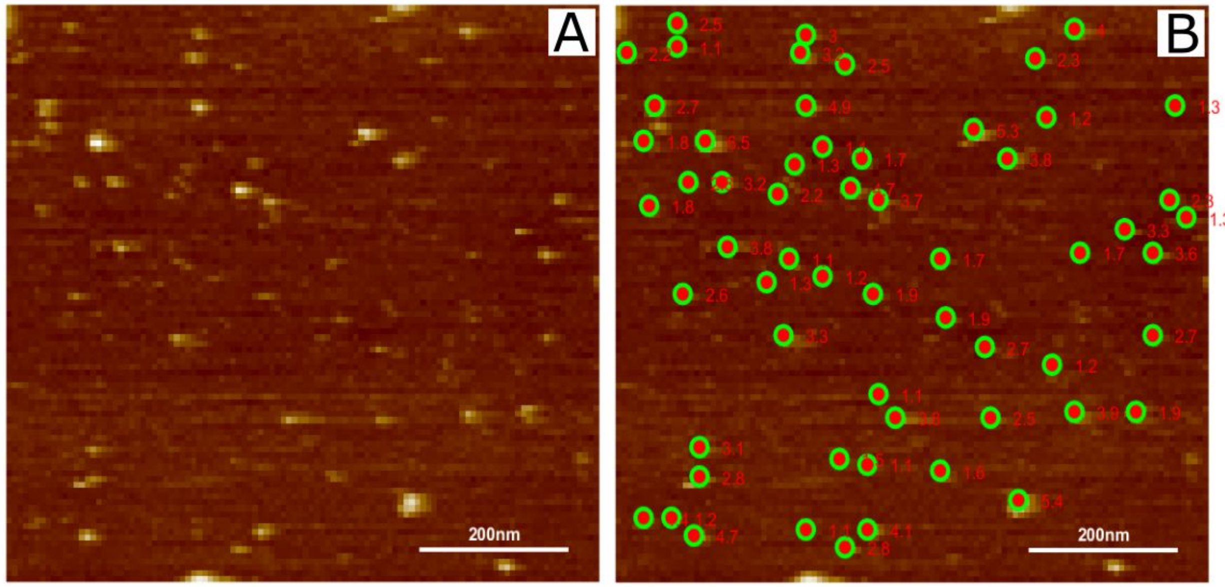

Figure S5: High-speed atomic force microscopy (HS-AFM) observation of S100A8 accumulation on mica surface. An image extracted from Video S2 at the 300-second time point is presented, with the selected area measuring 800 × 800 nm. Panel (A) depicts the morphology, while panel

(B) displays the height of individual S100A8 particles. The experiment was conducted in HEPES/NaOH buffer (pH 7.4) at a protein concentration of 10 µM.

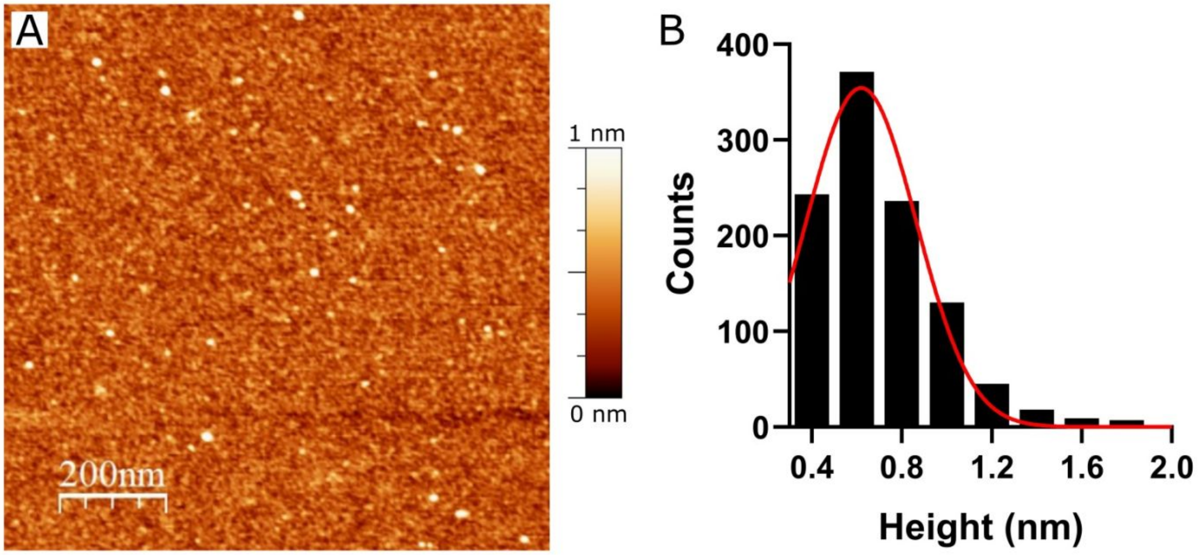

Figure S6: The morphology of the freshly purified S100A8 protein deposited on a mica substrate. Panel (A) shows an AFM topography image of the S100A8 protein, obtained with a scan area of 1 × 1 μm^2, a resolution of 512 × 512 pixels, and a scan speed of 0.8 Hz. The AFM image was acquired using a Dimension Icon AFM (Bruker, USA) operating in tapping mode. Silicon nitride probes (FESP, Bruker, USA) with a nominal spring constant of 2.8 N/m and a resonance frequency of 75 kHz were utilized, featuring a nominal tip radius of 8 nm. Panel (B) represents the Gaussian distribution for height, with a mean of 0.6 ± 0.2 nm (n = 1134).

Table S1: Size determination of S100A8-induced defects. The initial radii ($r_{0\ min}$) of preexisting defects and the S100A8-induced changes in radii (Δr) over time in DOPC/DOPE/DOPS/CHOL (2/3/3/2) solid-supported lipid bilayers, as presented in Figure 6, are detailed below. The average radius of the newly formed S100A8-induced defects (indicated in bold) is 25.9 ± 3.9 nm (n = 9).

Defect No.	$r_{0\ min}$ / nm	$\Delta r_{3\ min - 0\ min}$ / nm	$\Delta r_{12\ min - 3\ min}$ / nm
1	27.5	11.2	25.7
2	24.3	5.2	10.4
3	24.2	9.1	15.2
4	24.1	9.1	9.9
5	-	**25.7**	14.2
6	-	**24.2**	15.0
7	-	**24.4**	35.3
8	-	**21.0**	19.3
9	-	-	**32.7**
10	-	-	**23.5**

11	-	-	**24.9**
12	-	-	**32.1**
13	-	-	**24.8**

Table S2: Depth determination of S100A8-induced defects. The initial depth ($h_{0\ min}$) of preexisting defects and the S100A8-induced changes in depth (Δh) over time in DOPC/DOPE/DOPS/CHOL (2/3/3/2) solid-supported lipid bilayers, as presented in Figure 6, are detailed below. The average depth of the newly formed S100A8-induced defects (indicated in bold) is 0.9 ± 0.3 nm (n = 9).

Defect No.	$h_{0\ min}$ / nm	$\Delta h_{3\ min-0\ min}$ / nm	$\Delta h_{12\ min-3min}$ / nm
1	0.9	1.2	0
2	0.8	0.7	0.2
3	0.8	0.5	0.8
4	1.4	0.8	0
5	-	**0.8**	1.0
6	-	**0.7**	0.9
7	-	**1.0**	0.8
8	-	**0.6**	1.5
9	-	-	**1.6**
10	-	-	**0.7**
11	-	-	**0.9**
12	-	-	**1.2**
13	-	-	**0.8**

Noteworthy, a limitation of our study is that we were unable to detect initial defects smaller than 24 nm, the radius of the HS-AFM probe.

Supporting Videos:

Video S1: HS-AFM video of the S100A8 interaction with a BTLE lipid bilayer.

Video S2: HS-AFM video of the S100A8 interaction with a DOPC/CHOL lipid bilayer.

Video S3: HS-AFM video of the S100A8 interaction with a DOPC/DOPE/DOPS/CHOL lipid bilayer (single lipid patch).

Video S4: HS-AFM video of the S100A8 interaction with a DOPC/DOPE/DOPS/CHOL lipid bilayer.
